# Effect of quantity and intensity of pulsed light on human non-visual physiological responses

**DOI:** 10.1186/s40101-017-0137-7

**Published:** 2017-04-26

**Authors:** Qianying Dai, Yuria Uchiyama, Soomin Lee, Yoshihiro Shimomura, Tetsuo Katsuura

**Affiliations:** 1 Graduate School of Engineering, Chiba University, 1-33 Yayoicho, Inage-ku, Chiba, 263-8522 Japan; 2 Center of Environment, Health, and Field Science, Chiba University, 6-2-1, Kashiwanoha, Kashia, 277-0882 Japan

**Keywords:** Non-visual effect, Pulsed light, Quantity, Radiant flux, Power, Intensity, Irradiance, Pulse width, Pupillary constriction, Subjective evaluation, EEG, ipRGC

## Abstract

**Background:**

Exposure to pulsed light results in non-visual physiological responses in humans. The present study aims to investigate whether such non-visual effects are influenced to a greater extent by the intensity of lighting or by the power (quantity) of lighting.

**Methods:**

>Twelve healthy young male participants (23 ± 0.3 years, 21–24 age range) were recruited for the present study. Participants were exposed to light of varying levels of intensity and quantity whose frequency was held constant across the conditions, which consisted of exposure to blue (different intensity, constant quantity) and white (constant intensity, different quantity) LEDs. Pupillary constriction, electroencephalogram (EEG) alpha band ratio, subjective sleepiness, concentration and perception of blueness were measured.

**Results:**

Pupillary constriction and subjective concentration were significantly greater under the high-intensity and short pulse width (HS) condition than under the low-intensity and long pulse width (LL) conditions at three time points during exposure to high-intensity light. However, no significant differences were observed among the results at the three time points during exposure to different quantities of pulsed light.

**Conclusions:**

The results of the present study indicate that non-visual influences of pulsed light on physiological function are mainly determined not by the quantity but by the intensity of the emitted light, with relatively higher levels of intensity producing more significant physiological changes, suggesting potent excitation of intrinsically photosensitive retinal ganglion cells.

**Electronic supplementary material:**

The online version of this article (doi:10.1186/s40101-017-0137-7) contains supplementary material, which is available to authorized users.

## Introduction

Recent research has indicated that exposure to light results in non-visual physiological effects in humans [1–9], as well as visual effects (brightness or spectral distribution). Such research has resulted in the discovery of a third class of photoreceptors in the mammalian retina known as intrinsically photosensitive retinal ganglion cells (ipRGCs), which have a peak sensitivity to short-wavelength light of around 480 nm [10, 11]. Therefore, the strongest non-visual effects should result from exposure to blue light [10]. Indeed, research has indicated that exposure to blue light results in increased pupillary constriction [12], increased alertness [13–16], improved cognitive performance [17], suppression of melatonin [18] and phase advanced circadian rhythms [19].

Further, recent reports have revealed that intermittent pulses of light evoked greater non-visual responses than continuous light [20–22]. In our previous study, we mixed short pulses (100 μs) of blue light with white light and observed increased pupillary constriction even when participants could not perceive the light as blue [23]. This result suggests that such illumination, which participants perceive as white, may allow workers to maintain higher levels of arousal in an office setting.

The irradiance of light refers to the radiant flux (power or quantity: the product of pulse width and intensity) received by a surface per unit area (W/m^2^). The irradiance of pulsed light is often termed “intensity” (W/m^2^/s), though this term actually refers to the irradiance of a surface per unit frequency (W/m^2^/Hz or W/m^2^/s) or wavelength (W/m^2^/nm) in radiometry, leading to confusion with quantity or intensity of light (https://en.wikipedia.org/wiki/Irradiance). The aim of the present study was to investigate the non-visual effects of irradiance of pulsed light, in different levels of intensity and quantity via assessment of human pupillary constriction, electroencephalogram (EEG) alpha band ratio and subjective experiences of sleepiness in order to determine whether the intensity or quantity of light is the main factor influence non-visual physiological responses in humans under pulsed light conditions. The results of this analysis may further enhance our understanding of the non-visual effects of pulsed lighting and clarify the mechanisms underlying the differential responses for varying combinations of intensity and quantity of pulsed light.

We chose to analyse pupillary constriction for two reasons. First, measurements of pupillary constriction can be obtained relatively quickly, facilitating the testing of many stimulus combinations. Second, the amplitude and time course of pupillary constriction parallel to those of ipRGC photoresponses [24], suggesting that this behaviour can serve as a readout of ipRGC activity. That is, a stimulus inducing robust pupillary constriction may be inferred to strongly excite ipRGCs. We further evaluated the EEG alpha band, concentration and sleepiness in order to assess the non-visual effects of light on alertness [13–16] and cognitive performance [17], which have been reported to be associated with the activity of ipRGCs. Perceived blueness was also measured in order to distinguish whether the observed responses were related to the visual or non-visual effects of light.

## Methods

Twelve healthy males (23 ± 0.3 years, 21–24 age range) provided informed consent to participate in the present study. All participants were classified as having normal colour perception after completing the Farnsworth-Munsell 100 Hue Test [25]. Participants were instructed to avoid caffeinated beverages, alcohol and pain medication and to obtain seven consecutive hours of sleep one day prior to participation in the experiment. The present study was approved by the Bioethics Committee of the Graduate School of Engineering at Chiba University (No. 24–17).

**Fig. 1:**
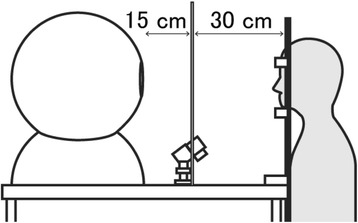
Layout of the experiment. An integrating sphere with light-emitting diodes (LEDs) was used as the lighting device, and the irradiance of lights was controlled by a computer programme. Participants sat on a chair with their heads facing a diffusion panel, which was located in front of the integrating sphere. Pupil diameter was measured in the left eye using an EMR-8 eye-tracking system with an infrared camera (Nac Image Technology, Inc., Japan)

Air temperature and relative humidity in the experiment room were controlled at 23 °C and 50%, respectively. An integrating sphere with light-emitting diodes (LEDs) was used as the lighting device. Participants sat on a chair with their heads facing a diffusion panel, which was located in front of the integrating sphere, as depicted in Fig. 1.

**Fig. 2:**
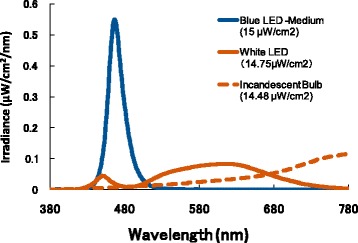
Spectral distribution curves. Spectral distribution curves for the blue LED, white LED, and incandescent bulbs. The peak wavelength of the pulsed blue light emitted by LEDs was 467 nm

An incandescent bulb (irradiance 14.48 μW/cm^2^; colour temperature 2524 K) was used as the base light, while the blue and white LEDs (irradiance 14.75 μW/cm^2^; colour temperature 2878 K) of the stimulus light source were arrayed in the integrating sphere. The spectral distribution curves for the blue LEDs, white LEDs, and incandescent bulb were using a spectroradiometer (HSR-8100, Maki Manufacturing Co., Ltd., California, USA). The spectral distribution curves are depicted in Fig. 2. The peak wavelength of the pulsed blue light emitted by LEDs was 467 nm.

**Fig. 3:**
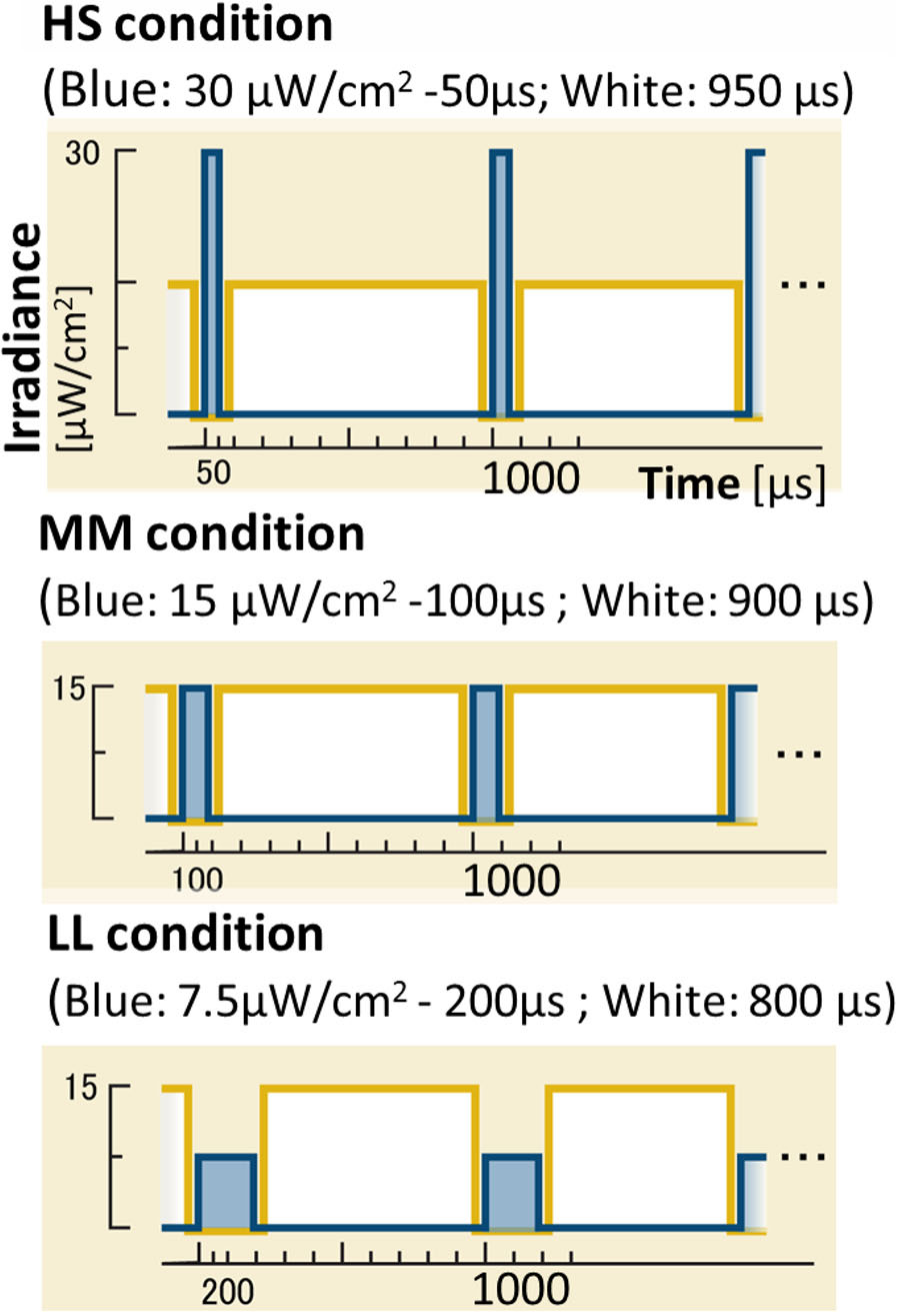
Three lighting conditions. Participants were exposed to three lighting conditions whose product was held constant. HS condition: high irradiance (30 kμW/cm^2^) and short pulse width (50 μs); MM condition: medium irradiance (15 μW/cm^2^) and medium pulse width (100 μs); LL condition: low irradiance (7.5 μW/cm^2^) and long pulse width (200 μs)

In the present study, in order to distinguish the effects of the intensity and quantity of pulsed light, we compared responses across three pulsed lighting conditions consisting of blue light (different intensity, constant quantity) and white light (constant intensity, different quantity) of equal frequencies (1 ms, 1000 Hz). The irradiance of all three conditions was measured at the participant’s eye level, as depicted in Fig. 3. Three blue pulsed lighting conditions with constant quantity (products of both intensity and pulse width) were implemented as follows: high intensity (30 μW/cm^2^) and short pulse width (50 μs) (HS condition); medium intensity (15 μW/cm^2^) and medium pulse width (100 μs) (MM condition); low intensity (7.5 μW/cm^2^) and long pulse width (200 μs) (LL condition). White pulsed light conditions of equal intensity (14.75 μW/cm^2^) were implemented as follows: long pulse width (950 μs) (HS condition); medium pulse width (900 μs) (MM condition); short pulse width (800 μs) (LL condition).

The intensity of blue light was highest in the HS condition, followed by the MM condition and reaching its lowest value in the LL condition. The quantity of white light was the highest in the condition HS, followed by the MM condition and reaching its lowest value in the LL condition. Longer durations of exposure to pulsed light were associated with larger differences in quantity among the three light conditions, such that the quantity was highest after 8 min than after 4 min, which was higher than that after 5 s. All light conditions resulted in light of equivalent frequency (1 ms, 1000 Hz). The pulsed blue and white lights of the stimulus light source were turned on and off in a constant, alternating fashion, with light pulses occurring every 1 ms (1000 Hz).

The characteristics of each pulsed light condition at the participant’s eye level for the three light conditions are shown in Table 1. The irradiance was calculated in the range of 380 to 780 nm. The melanopsin-stimulating irradiance and photon density at the retinal level during each light condition are also described in Table 1. Melanopsin-stimulating irradiance and photon density were estimated [26] based on the spectral absorption of the crystalline lens [27] and a template [28] indicating the spectral absorption characteristics of the photopigment with a peak wavelength of 484 nm [10].

**Table 1:** Characteristics of each light condition

Condition	HS condition		MM condition		LL condition		Incandescent
Light-emitting diode	Blue light	White light	Blue light	White light	Blue light	White light	
Condition	High intensity Short pulse width	Long pulse width	Medium intensity Medium pulse width	Medium pulse width	Low intensity Long pulse width	Short pulse width	Incandescent
Irradiance (μW/cm2)	30 μW/cm^2^	14.75 μW/cm^2^	15 μW/cm^2^	14.75 μW/cm^2^	7.5 μW/cm^2^	14.75 μW/cm^2^	14.48 μW/cm^2^
Pulse width (μs)	50 μs	950 μs	100 μs	900 μs	200 μs	800 μs	1000 μs
Peak wavelength (nm)	467 nm	–	467 nm	–	467 nm	–	–
Photopic illuminance (lx)	24.00	44.00	12.23	44.00	6.00	44.00	16.00
Scotopic illuminance (lx)	351.00	53.00	176.00	53.00	88.00	53.00	20.00
Photon density (1012 photons/cm^2^/s)	72.12	44.35	36.06	44.35	18.03	44.35	49.72
Photon density (log photons/cm^2^/s)	13.86	13.65	13.56	13.65	13.26	13.65	13.70
Melanopsin-stimulating intensity (μW/cm^2^)	24.65	2.41	12.32	2.41	6.16	2.41	0.97
At retinal level products of melanopsin-stimulating irradiance and pulse width per 1 ms (μW/cm^2^)	1.23	2.29	1.23	2.17	1.23	1.93	0.97
Summation of products of blue and white pulsed light per 1 ms (μW/cm^2^)		3.52		3.40		3.16	–

### Study protocol

**Fig. 4:**
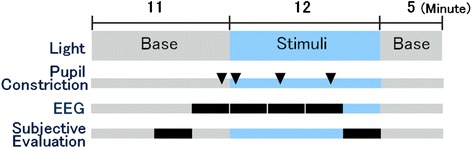
Experiment protocol. Participants rested for 11 min under the base lighting condition (15 μW/cm^2^; 2524 K) prior to exposure to each of the three stimulus lighting conditions. In addition to measuring pupillary constriction, EEG alpha band ratio and subjective evaluations were obtained before and during exposure to the three stimulus lighting conditions

The experimental protocol is summarised in Fig. 4. Participants were instructed to sit quietly at rest and maintain the same posture in order to ensure similar exposure during each lighting condition. Participants rested for 11 min under the base lighting condition (15 μW/cm^2^; 2524 K) prior to exposure to stimulus lighting conditions. In addition to measuring pupillary constriction and EEG alpha band ratio (8–13 Hz), we assessed subjective sleepiness and concentration using the Visual Analogue Scale (VAS). Further assessment of subjective sleepiness was conducted using the Kwansei Gakuin Sleepiness Scale (KSS). The KSS estimates wakefulness according to the presence of sleep symptoms and is often used in conjunction with the VAS assessment of sleepiness and other physiological indices for more effective overall evaluation of sleepiness [29, 30]. Participants were also asked to report subjective blueness for each light condition. Changes from before and during exposure to stimulus lighting conditions were also recorded. Each stimulus lighting condition lasted 12 min. In order to ensure sufficient recovery of pupil diameter, an interval of approximately 5 min was included between successive stimulus lighting conditions. The three stimulus lighting conditions were performed at approximately the same time on two different days. The order of the three lighting conditions was counterbalanced among the participants.

Pupil diameter of the left eye was measured using an eye-tracking system (EMR-8; NAC Image Technology Inc., Japan), and the extent of pupillary constriction was calculated as follows (Formula 1):

1 $$ \begin{array}{c}\hfill \mathrm{percentage}\;\mathrm{of}\;\mathrm{pupil}\mathrm{lary}\;\mathrm{constriction}\;\left(\%\right)=\hfill \\ {}\hfill\;\left[\left(\mathrm{baseline}\;\mathrm{pupil}\;\mathrm{diameter}\hbox{--} \mathrm{pupil}\;\mathrm{diameter}\;\mathrm{during}\;\mathrm{stimulus}\;\mathrm{lighting}\;\mathrm{condition}\right)/\mathrm{baseline}\;\mathrm{pupil}\;\mathrm{diameter}\right]\hfill \\ {}\hfill \times 100.\hfill \end{array} $$

Baseline pupil diameter was recorded as the mean value obtained 20 s prior to pulsed light exposure. Pupil diameters for the stimulus lighting conditions were recorded as the mean value obtained over 20 s following an initial exposure period of 5 s and then again after 4 and 8 min.

Based on the international 10–20 system for electroencephalography, three EEG electrode locations were selected for evaluation of the non-visual effects of light on alertness: Fz (frontal region), Cz (central region) and Pz (occipital region). EEG activity was recorded at the Fz, Cz and Pz electrode sites using the linked earlobe electrodes as a reference. EEG signals were amplified using a multichannel bioamplifier (MP150 system, BIOPAC Systems, California, USA) with a time constant of 0.3 s using a low-pass filter of 35 Hz and a high-pass filter of 0.05 Hz. Alpha band signals were defined as those falling within the 8–13 Hz frequency band. Changes in alpha band signals under stimulus lighting conditions were calculated relative to those obtained under base lighting conditions.

The VAS assessments for subjective evaluation of concentration, sleepiness and perception of blueness, as well as the KSS, were conducted both prior to and following the experiment, as indicated in Fig. 4. Participants were asked to report their “perceived level of blueness” after the 8-min point during the initial exposure period along a VAS with “Does not seem blue” and “Seems very blue” at opposite ends of a 10-cm line. Participants pointed out the level of perceived blueness along the line, and the subjective results were digitised after the experiment. Changes in these parameters were also calculated relative to values obtained under base lighting conditions.

One-way repeated-measures analysis of variance (ANOVA) (SPSS version 11, IBM Analytics, Illinois, United States) was used to examine the effect of lighting condition on pupillary constriction and EEG activity, as well as subjective parameters. A significance level of 0.05 was used in all comparisons.

## Results

**Fig. 5:**
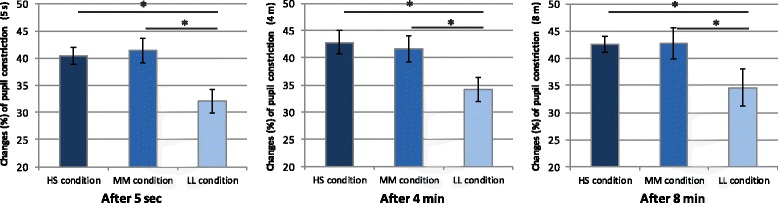
Changes (%) in pupillary constriction under three light conditions (mean ± SE). Average changes (%) in pupillary constriction after exposure to stimulus lighting 5 s. Greater pupillary constriction was observed under the high- and medium-irradiance conditions than under the low-irradiance condition. *p < 0.05

Table 2 shows the results for pupillary constriction and other subjective indices obtained during the three light conditions. Significantly greater changes were observed in pupillary constriction under both the HS (Blue 30 μW/cm^2^; 50 μs) and MM (Blue 15 μW/cm^2^; 100 μs) conditions at the 5-s and 4- and 8-min points following initiation of the stimulus lighting condition than under the LL condition (Blue 7.5 μW/cm^2^; 200 μs) (*p* < 0.05) (Fig. 5). No significant differences were observed in pupillary constriction among the three time points under each lighting condition.

**Table 2:** Pupillary constriction and subjective indices during the three light conditions

Condition		HS condition	MM condition	LL condition
Pupillary constriction				
After exposure 5 s	Mean	40.45*	41.37*	32.10
	SD	5.31	7.14	6.80
After exposure 4 min	Mean	42.87*	41.69*	34.13
	SD	6.66	7.51	7.06
After exposure 8 min	Mean	42.61*	42.70*	34.62
	SD	4.68	9.03	10.78
Blueness (VAS)	Mean	57.74	54.41	51.29
	SD	18.05	23.27	19.33
Concentration (VAS)	Mean	27.30*	3.76	1.72
	SD	20.99	18.85	17.31
KSS	Mean	−0.02	0.10	−0.03
	SD	0.15	0.27	0.14
Sleepiness (VAS)	Mean	−0.05	0.08	0.34
	SD	0.31	0.50	0.51

*SD* standard deviation

*p < 0.05 compared with LL condition

**Fig. 6:**
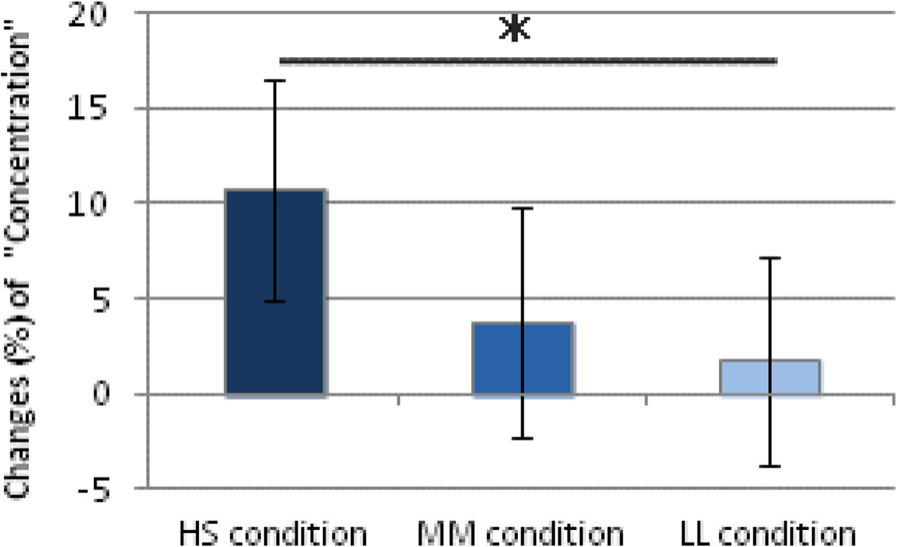
Changes (%) in subjective concentration under three light conditions (mean ± SE). Changes (%) in subjective concentration under the high-irradiance condition were significantly larger than under the low-irradiance condition. *p < 0.05

**Fig. 7:**
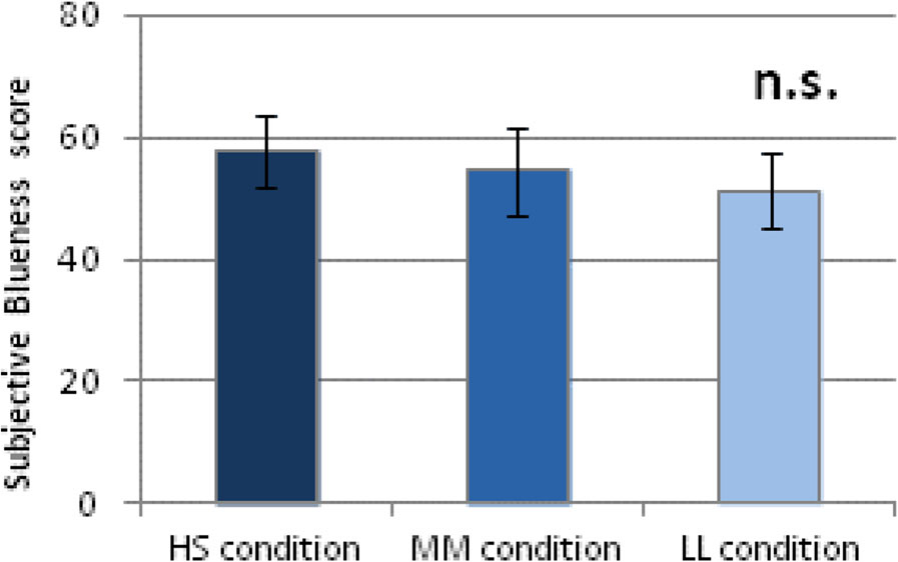
Subjective blueness score under three lighting conditions. No significant differences (n. s.) in perception of blueness were observed

Changes in subjective concentration scores were significantly larger under the HS (Blue 30 μW/cm^2^; 50 μs) condition than under the LL (Blue 7.5 μW/cm^2^; 200 μs) condition (*p* < 0.05) (Fig. 6). No significant differences in perception of blueness were observed among the three pulsed lighting conditions (Fig. 7). Further, no significant effects of light condition were observed with respect to KSS score, subjective sleepiness.

Table 3 shows the EEG alpha band ratio results obtained during the three light conditions at each time point. No significant differences in EEG alpha band ratio were observed in the three selected regions of the brain at any time point.

**Table 3:** EEG alpha band ratio during the three light conditions

Condition		HS condition	MM condition	LL condition
Cz				
After exposure 5 s	Mean	−0.08	−0.08	−0.08
	SD	0.06	0.07	0.06
After exposure 4 min	Mean	−0.09	−0.10	−0.09
	SD	0.08	0.08	0.09
After exposure 8 min	Mean	−0.09	−0.10	−0.09
	SD	0.10	0.08	0.08
Fz				
After exposure 5 s	Mean	−0.09	−0.09	−0.10
	SD	0.07	0.07	0.07
After exposure 4 min	Mean	−0.09	−0.10	−0.11
	SD	0.08	0.09	0.09
After exposure 8 min	Mean	−0.11	−0.11	−0.10
	SD	0.10	0.09	0.08
Pz				
After exposure 5 s	Mean	−0.06	−0.06	−0.05
	SD	0.04	0.05	0.05
After exposure 4 min	Mean	−0.08	−0.07	−0.06
	SD	0.07	0.06	0.08
After exposure 8 min	Mean	−0.08	−0.08	−0.07
	SD	0.09	0.08	0.07

*SD* standard deviation

*p < 0.05 compared with LL condition

## Discussion

The present study aimed to investigate whether non-visual effects of pulsed light are influenced to a greater extent by the intensity of lighting or by the quantity of lighting. Since this aim could not be attained using the results of a two-factor ANOVA that utilises intensity and quantity as independent factors, we examined the effects of different levels of intensity and quantity during three lighting conditions using a one-way repeated-measures ANOVA, with the frequency of pulsed light held constant at 1000 Hz.

In the present study, we observed significantly different effects of varying light conditions on pupillary constriction and subjective concentration even when the quantity of pulsed blue light remained constant across conditions. However, no significant difference was observed in the subjective perception of blueness under the three pulsed stimulus lighting conditions, suggesting that differential non-visual effects of light exposure may occur even when participants perceive no differences in the colour of blue light emitted.

We also observed that, although the three pulsed blue light conditions maintained an equal product of irradiance and pulse width at all three time points, greater pupillary constriction was observed under the high- and medium-intensity conditions than under the low-intensity condition. Previous studies have suggested that the pupillary response to light is controlled by rods in conditions of low irradiance but by ipRGCs under conditions of high irradiance [12, 31]. For example, Panda et al. [31] reported that ipRGCs contribute to the pupillary response in mice at an irradiance level greater than approximately 13 log photons/cm^2^/s when animals are exposure to 470-nm light at eye level. Takahashi et al. [12] also estimated that the irradiance level at which the role of rods in pupillary constriction is replaced by ipRGCs is approximately 10.4 × 10^12^ photons/cm^2^/s when participants are exposed to eye-level 457-nm light. They further reported that the threshold retinal irradiance for depolarization of ipRGCs in rats is approximately 12.7 log photons/cm^2^/s of 500-nm light at eye level [12]. In the present study, only melanopsin-stimulating irradiance and photon density of blue pulsed light were equivalent in the HS condition (72.1 × 10^12^ or 13.9 log photons/cm^2^/s), MM condition (36.1 × 10^12^ or 13.6 log photons/cm^2^/s), and LL condition (18.0 × 10^12^ or 13.3 log photons/cm^2^/s). Levels of 467 nm of pulsed blue light were high enough to result in increased pupillary constriction associated with ipRGCs rather than rods, which controls the pupillary response under conditions of low irradiance.

Perhaps most interesting is the finding that, although the difference in exposure quantity was largest at the 8-min point during the HS condition than that in the MM and LL conditions at 4-min and 5-s points, no significant differences in pupillary constriction were observed among the three time points. We therefore propose that the non-visual effects of light on pupillary constriction, which occur via stimulation of ipRGCs, are determined by exposure to relatively higher intensities of lighting, but not determined by exposure to relatively larger quantities of pulsed light. Do et al. reported that the density of ipRGCs in the retina is extremely low compared to that of cones and rods, resulting in a very low photon catch. However, another study has indicated that the higher and prolonged responses of ipRGCs to single photon may be responsible for these results [32], consistent with our findings.

Prolonged exposure to intense light may lead to damage of the retinal photoreceptors [33]. According to results of the present study, reducing the pulsed width of intermittent lighting may make phototherapy safer. Continued research in this field may help to generate new methods for both therapeutic and functional lighting.

## Conclusion

In the present study, participants were exposed to three pulsed light conditions in which the frequency remained equal for varying levels of intensity and quantity of pulsed light. Significant differences were observed in non-visual effects (e.g., pupillary constriction and subjective concentration) even though participants reported no differences in their perception of the light’s blueness, suggesting that the mechanisms underlying the visual and non-visual physiological responses to light are different.

Furthermore, exposure to pulsed light of higher intensity and shorter pulsed width resulted in significant increases in pupillary constriction after 5 s even when the quantity (product of irradiance and pulse width) of blue pulsed light was held constant. However, exposure to pulsed light with larger differences in quantity after 8 min resulted in no significant increase in pupillary constriction relative to that at the 5-second and 4-min points when the intensity of light was constant.

The results of present study indicate that, relatively higher intensity and shorter pulsed-widths of blue light (HS condition, after 5 s) produced a more significant influence on ipRGCs and physiological functions even when the quantity of blue pulsed light are held constant. Relatively larger differences in the quantity of white pulsed light associated with longer exposure time (HS condition, after 8 min) produced no significantly different influence on ipRGCs and physiological functions when compared with HS conditions at the 5-s and 4-min points. These results suggest that the intensity of pulsed light, rather than the quantity, is the main factor influencing ipRGCs and physiological functions.

With regard to more practical applications, our results indicate that it is possible for light to exert two effects at the same time. That is, the increased intensity of blue light may produce more significant physiological changes and non-visual influences on ipRGCs, while reduced quantity of pulsed light may make phototherapy safer and more energy efficient.

## Additional files

Additional file 1: Lighting spectral distribution data. Spectral distribution data for the blue LED, white LED, and incandescent bulbs. (XLSX 47 kb)

Additional file 2: Results data. Significant results for pupillary constriction, subjective blueness, and concentration date under the three lighting conditions. (XLSX 54 kb)

## Abbreviations

ANOVA: Analysis of variance
EEG: Electroencephalography
ipRGC: Intrinsically photosensitive retinal ganglion cell
KSS: Kwansei Gakuin Sleepiness Scale
LED: Light-emitting diode
VAS: Visual Analogue Scale
